# The Effects of Rocuronium-sugammadex on Fetomaternal Outcomes in Pregnancy Undergoing Electroconvulsive Therapy: A Retrospective Case Series and Literature Review

**DOI:** 10.7759/cureus.4820

**Published:** 2019-06-03

**Authors:** Mahmut A Karahan, Evren Büyükfırat, Orhan Binici, Hacer Uyanıkoğlu, Adnan Incebıyık, Mehmet Asoğlu, Nuray Altay

**Affiliations:** 1 Anesthesiology and Critical Care, University of Harran, Sanliurfa, TUR; 2 Obstetrics and Gynecology, University of Harran, Sanliurfa, TUR; 3 Psychiatry, University of Harran, Sanliurfa, TUR

**Keywords:** pregnancy, electroconvulsive therapy, anesthesia, psychiatry

## Abstract

Introduction

The aim of this study was to evaluate the anesthesia management of pregnant patients who received electroconvulsive therapy (ECT) at our hospital and to examine the effects of this procedure on mothers and fetuses.

Methods

This study was conducted with 15 pregnant patients who underwent the ECT procedure who did not benefit from medical treatment or who did not want medical treatment. We evaluated the psychiatric, obstetric, and especially anesthesiology records of these patients. All of the patients received 1 mg/kg propofol with 0.6 mg/kg rocuronium. Eight mg/kg sugammadex was used to terminate the effects of the non-depolarizing neuromuscular blocking agents. Their demographic characteristics, history of diagnosis, total ECT sessions, duration of hospitalization, discharge status, neonatal outcomes, short- and long-term maternal or fetal complications, anesthetic management, and recovery parameters were retrospectively reviewed.

Results

Fifteen pregnant patients received a total of 95 ECT treatments. No anesthesia-related maternal complications developed. In terms of the recovery parameters of the patients, the mean duration of the motor seizure was 28.7 ± 6.3 seconds, the mean time to spontaneous respiration was 224 ± 21.8 secs, the mean time to opening the eyes was 403.6 ± 21.1 secs, and the mean time to command compliance was 415.24 ± 81.15 secs. The mean gestational week was 14.06 ± 6.65, and the mean number of pregnancies was 2.87 ± 2.29. Seven (46,7%) patients were in the first trimester of pregnancy, six (40%) were in the second trimester, and two (13.3%) were in the third trimester. Spontaneous abortion occurred in four patients, six patients gave birth by spontaneous vaginal delivery, and five patients delivered by cesarean section. Neonatal respiratory distress developed in only one fetus.

Conclusion

Anesthesia management during ECT can be provided safely by using propofol and rocuronium-sugammadex in pregnancy in the postoperative period. However, there is a risk of abortion and neonatal respiratory distress in the use of ECT, especially in the first trimester period. It is advisable to inform the patient's family in detail before this procedure outcome.

## Introduction

Obstetric patients are generally young, healthy females. Pregnancy involves physical, psychological, and physiological changes as it progresses. The pregnancy and postpartum periods pose many risks to women with psychiatric disorders, especially depression, as the symptoms of such conditions worsen during these experiences [1-3]. Psychiatric disorders that emerge or recur during pregnancy can cause serious problems for both the mother and the fetus [4-6]. However, pharmacotherapy carries the risk of major congenital malformations in the first trimester of pregnancy and of drug toxicity or withdrawal effects in the third trimester. In this context, electroconvulsive therapy (ECT) is a rapid and effective treatment option for cases of advanced major depression, bipolar disorder, anxiety disorders, and psychotic disorders [7-8]. ECT, which creates generalized convulsions by stimulating the brain tissue with an electric current, is an effective treatment for psychiatric disorders. These requirements for anesthesia, together with the primary requirements of amnesia and muscle relaxation, require control of hemodynamic changes and related complications [9]. Recent developments in anesthesia techniques have increased the efficacy and reliability of ECT. However, the optimum anesthetic drug or combination thereof has yet to be identified. As ECT is a brief procedure; the anesthetics used should have an effect over a short duration, enable rapid recovery, and not harm the mother or fetus [10].

Due to the recent increase in the prevalence of psychiatric disorders and the more frequent use of ECT, anesthetists are encountering more pregnant recipients of ECT, rendering issues related to anesthesia management of even greater importance. The aim of this study was to evaluate the anesthesia management of pregnant patients who received ECT and to examine the effects of this procedure on mothers and fetuses.

## Materials and methods

Patients

The hospital’s electronic records system was scanned for the period from January 2016 to February 2017, and we retrospectively evaluated the psychiatric, obstetric, and anesthesiology records of 15 pregnant patients who were treated with ECT.

Clinical outcome

Neonatal records of the offspring of the patients who had given birth were reviewed and the patients were interviewed by telephone to retrieve any missing information such as the gestational week of birth, mode of delivery, and health status of the neonate.

Data on demographic characteristics, gestational week, parity, psychiatric diagnosis, motor seizure duration, ECT duration, duration of anesthesia, time to return of spontaneous respiration, time to opening eyes, recovery time from administration of the sedative until compliance with simple commands, comorbid conditions, anesthetic medications, postoperative complications, the length of hospital stay, modified Aldrete score (MAS), and total number of ECT applications were recorded for each patient.

ECT procedure

In our hospital, ECT is usually delivered with very short pulsating using a Thymatron System II bipolar ECT device (Somatics LLC, IL, US). Each application involves a 30- to 60-second seizure. Bilateral, bitemporal ECT was applied in each patient. The maximum charge of the device is 1004 millicoulumb (mC) at a 100% dose, in which the charge applied to each patient is expressed as a percentage of the electrical dose applied to the patient. The first seizure threshold was defined based on the “half-age method.” In this method, half of the patient's age is taken as the base value in percentage. During the ECT application, seizures were monitored via electroencephalography (EEG) data of the device.

Anesthetic management

A preoperative anesthetic examination was performed before the ECT procedure in each patient. General anesthesia was induced by using propofol as a hypnotic-sedative agent and rocuronium as a neuromuscular block and a muscle relaxant, with a dose of 1 mg/kg propofol with 0.6 mg/kg rocuronium. Additionally, 8 mg/kg sugammadex was administered to terminate the effects of the non-depolarizing neuromuscular blocking agents. The patients were ventilated manually with 100% oxygen 3 L/min using a face mask.

Obstetric protocol

An obstetric examination was performed in each patient by the obstetrics and gynecology department immediately before and after each ECT procedure. Fetal heart rate (FHR) monitoring was performed before and after ECT with a Doppler monitor. Patients were monitored with tocometry for uterine activity.

Statistical analysis

Data were analyzed using SPSS for Windows v 20.0 software (IBM Corp., Armonk, NY, US). Descriptive statistics for quantitative data were expressed as mean ± standard deviation (SD).

## Results

Electroconvulsive therapy (ECT) was administered 486 times in 81 female patients over the period between January 1, 2016, and February 1, 2017. Of these, 15 patients were pregnant and received a total of 95 ECT treatments. The mean number of ECT procedures per patient was 6.33±3.77. ECT was applied in five (33.3%) patients with bipolar disorder, nine (60%) patients with major depression, and one (6.7%) patient with unspecified psychosis. Of the 15 patients, two (40%) of the patients with bipolar disorder and five (55.55%) of the patients with depression received their first diagnosis. In total, 65 ECT procedures were administered in patients diagnosed with depression, 28 procedures in the patients with bipolar disorder, and two procedures in the one patient with unspecified psychosis. One of the patients diagnosed with depression also had comorbid hypothyroidism and another patient had asthma.

The mean age of the patients was 29.46±5.17 years, the mean gestational age was 14.06±6.65 weeks, and the mean number of pregnancies was 2.87±2.29. Seven (46.7%) patients were in the first trimester of pregnancy, six (40%) were in the second trimester, and two (12.3%) were in the third trimester (Table 1). Nine patients had been previously diagnosed and all of them had stopped taking medication after the onset of pregnancy. Spontaneous abortion occurred in four patients, six patients gave birth by spontaneous vaginal delivery, and five patients gave birth by cesarean section.

**Table 1:** Demographic and clinical characteristics of the patients

	Mean±SD, n (%)
Age (years)	29.46±5.17
Mean gestational age (weeks)	14.06 ± 6.65
First Trimester	7 (46.7%)
Second Trimester	6 (40%)
Third Trimester	2 (13.3%)
Average parity	2.87 ± 2.29
Patients with accompanying diseases	
	Hypothyroidism (n=1)
	Asthma (n=1)
Diagnosis	
Depression	9 (60%)
Bipolar Disorder	5 (33.3%)
Psychosis	1 (6.7%)
Length of hospital stay (days)	19 ± 9.02
Discharge Status	
Completely improved	6 (40%)
Significantly improved	6 (40%)
Left treatment at her own will	3 (20%)

One infant that was delivered by cesarean section developed neonatal respiratory distress. The five cesarean deliveries were performed by three patients with bipolar disorder and two patients with depression; all of these patients were administered general anesthesia. Although general anesthesia is not a routine procedure in such patients, we performed general anesthesia since the patients refused to undertake local anesthesia. Cesarean sections were performed in two of the patients with bipolar disorder (manic episode) due to a placental anomaly in one patient and fetal stress in the other. The other patients who underwent cesarean section had previously given birth by cesarean section. The mean length of hospital stay based on the ECT procedure was 19±9.02 days for all patients (Table 2).

**Table 2:** Treatment summary ECT: Electroconvulsive Therapy

Patient number	Gestational Week	Diagnosis	Total ECT Sessions	Gestational Week	Mode of Delivery	Maternal or fetal Complication
#1	20	Bipolar disorder (Manic Episode)	4	17	Normal vaginal	None
#2	12	Major depression	6	28	Cesarean section	Neonatal respiratory distress
#3	9	Unspecified psychosis	2	7	Normal vaginal	None
#4	16	Major depression	3	10	Normal vaginal	None
#5	8	Major depression	3	6	Abortion	Abortion
#6	8	Bipolar disorder (Depression Episode)	2	6	Abortion (12 GW)	Abortion
#7	6	Major depression	7	19	Abortion	Abortion
#8	14	Major depression	7	17	Normal vaginal	None
#9	14	Major depression	11	32	Normal vaginal	None
#10	6	Major depression	5	20	Cesarean section	None
#11	28	Major depression	13	21	Normal vaginal	None
#12	16	Bipolar disorder (Manic Episode)	13	22	Cesarean section	None
#13	26	Bipolar disorder (Depression Episode)	4	26	Cesarean section	Placental abruption
#14	16	bipolar disorder (Manic Episode)	5	19	Cesarean section	None
#15	12	Major depression	10	35	abortion	Abortion

No anesthesia-related maternal complications occurred in any patient. In terms of the recovery parameters, the mean duration of motor seizure was 28.7±6.3 s, the mean time to spontaneous respiration was 224 ± 21.8 s, mean time to opening eyes was 403.6 ± 21.1 s, and mean recovery time from administration of the sedative until compliance with simple commands was 415.24 ± 81.15 s. The mean time to reach a MAS score of 9, which is required for the transfer of the patient to the psychiatry clinic, was 499.1 ± 45.3 s (Table 3).

**Table 3:** Anesthetic recovery parameters MAS: Modified Aldrete Score

Recovery parameters	Mean±SD,
Mean time to spontaneous breathing	224 ± 21.8 sec
Mean time to opening eyes	403.6 ± 21.1 sec
Mean time to command compliance	415.24 ± 81.15 sec
Mean duration of the motor seizures	28.7 ± 6.3 sec
Time to reach MAS 9	499.1 ± 45.3 sec
Complications	None

## Discussion

Electroconvulsive therapy (ECT) has been traditionally performed under anesthesia for the last 60 years, and anesthesia is now an indispensable part of the ECT procedure. In addition to traditional approaches, alternative anesthetic approaches have been developed, particularly due to rapid changes in the development of anesthetic drugs within the last decades. With the positive developments in anesthetic techniques, drugs, and teams, ECT applications have brought a different approach to the treatment of pregnant patients. However, studies reporting on the effects of drugs used in anesthetic practice on both mother and fetus are very limited.

Literature reviews indicate that there is little information on the combined use of rocuronium, sugammadex, and propofol in ECT applications. Moreover, to our knowledge, there is no study reporting on the side effects of this combination. Our study is the first to evaluate fetomaternal complications in both the short- and long-term use of the combination of rocuronium, sugammadex, and propofol in ECT applications.

When ECT is administered in pregnant women, the ideal anesthetic agents should have a rapid onset, should not affect hemodynamics, and should not shorten the duration of the seizure due to the physiological changes that occur in pregnancy and the presence of the fetus. Additionally, the effect should be rapidly terminated. The anesthetic agents used for ECT application in this study may have potential embryonic risks. However, none of the agents we used for premedication or induction during the ECT procedure was in pregnancy category A. Different anesthetic agents, such as methohexital, sevoflurane, thiopental, and propofol, have been used for anesthetic induction when administering ECT in pregnant women. The two most commonly used anesthetics during pregnancy include methohexital and propofol [6,11]. Propofol differs from other anesthetic agents since it achieves rapid induction and recovery, reduces the risk of nausea and vomiting, and suppresses the hemodynamic response. It is also rapidly removed from fetal circulation due to its low molecular weight and low solubility in fat. Therefore, propofol can be considered as an ideal agent for ECT during pregnancy [5,12]. Meaningfully, recent reports have indicated that propofol has been the most commonly used agent in anesthetic induction in pregnant women undergoing ECT [5,9,13-14]. In a case report by De Asis et al., ECT was applied at the 23rd gestational week in a patient with bipolar disorder. Moreover, although methohexital and succinylcholine were initially used for anesthetic induction, the drugs were replaced with propofol since fetal bradycardia developed following fetal deceleration as a result of prolonged seizures. The authors also noted that the duration of seizures was shorter with propofol and the fetus was protected in a stable state [15]. Both recent reports and the data from our clinical practice indicate that 1 mg/kg propofol is often administered for anesthetic induction in ECT application.

Neuromuscular blocking agents are used for restricting motor activity during ECT. Succinylcholine is the preferred depolarizing muscle relaxant, as it has a short-term effect [5]. Non-depolarizing neuromuscular blocking agents, another group of neuromuscular blocking agents, are generally used instead of succinylcholine in patients with different comorbidities. Unlike succinylcholine, these medications do not lead to serious side effects, such as myalgia, headache, intragastric pressure, increased intraocular pressure, risk of malignant hyperthermia, or hyperkalemia [16]. Rocuronium, which is increasingly used in ECT as an alternative to succinylcholine, is a neuromuscular blocking agent with a steroidal structure, which has an effect of moderate duration and is characterized by nondepolarizing properties. Sugammadex is a new-generation recovery agent used for terminating the effect of nondepolarizing neuromuscular blocking agents. As rocuronium has a rapid onset and its effect is rapidly recovered with sugammadex, there has been an increasing trend in recent years toward its use as an appropriate alternative to succinylcholine, which is the most important indication suggesting that rocuronium is a good alternative to succinylcholine [17-19].

Studies investigating the effects of rocuronium, sugammadex, and succinylcholine on recovery from ECT have revealed that the times to reaching a score of MAS 9, opening eyes, command compliance, and spontaneous respiration were shorter in patients administered with the combined use of rocuronium and sugammadex [20]. Another study, which used succinylcholine and rocuronium as muscle relaxants during ECT, administered different doses of sugammadex and compared the recovery times. The study indicated that the efficacy of 0.6 mg/kg rocuronium with 8 mg/kg sugammadex was similar to that of 1 mg/kg succinylcholine and the spontaneous recovery times were also similar [21]. The present study administered 0.6 mg/kg rocuronium with 8 mg/kg sugammadex and the time to return of spontaneous breathing was consistent with the findings of previous studies.

The risk of gastric reflux and aspiration pneumonia is higher in the last trimester of pregnancy. However, although our patients were in early pregnancy, we recommended six-hour fasting and antacids for all patients before the procedure. To prevent aortocaval compression that could reduce placental perfusion, we placed a wedge under the patients' right hip. ECT may be a safe procedure in pregnant patients; however, airway management may not be safe. Airway management is usually provided by a face mask in such cases since the majority of the cases have a low gestational week and a short duration of processing, mainly due to the short duration of the effects of neuromuscular blocking agents used and the early return of spontaneous breathing [5,13-14]. In our study, we used the aspiration measures mentioned above and the patients were ventilated manually with 100% oxygen 3 L/min using a face mask. Due to the measures mentioned above, no peri- or postoperative maternal complications occurred in any patient because of the combined use of rocuronium and sugammadex, which allowed rapid compilation with the short-acting propofol we used as an induction agent in anesthesia. Instead, the literature consists primarily of retrospective studies and case reports. However, to the best of our knowledge, there has been no ECT-related mortality among pregnant patients undergoing ECT. Nevertheless, it seems that ECT may be associated with certain problems that arise during pregnancy such as vaginal bleeding, uterine spasm, abdominal pain, miscarriage, placental abruption, pre-eclampsia, and premature birth by cesarean section. In our study, placental abruption occurred in only one patient who received ECT at 26 weeks of gestation in the late follow-up period, underwent cesarean section at 39 weeks of gestation, and had no fetal anomaly. In four recent systematic reviews, abruptio placentae was identified in one case in each study [22].

Accordingly, we consider that placental abruption may result from the ECT procedure since the gestational age is advanced in such patients [23]. The number of cesarean sections in our study differs from those reported by each of the four recent systematic reviews. In our study, the five cesarean deliveries were performed in three patients with bipolar disorder (manic episode) and two patients with depression. Of the cases with bipolar disorder, one patient underwent cesarean section due to a placental anomaly and the other one due to fetal stress. The other patients who underwent cesarean delivery had previously given birth by cesarean section. Overall, the number of patients that underwent cesarean section is higher than those reported in the literature. On the other hand, no complications such as uterine contractions, abdominal pain/pelvic pain, or preterm vaginal bleeding occurred in any of our patients.

Although there is no evidence suggesting that ECT does not lead to fetal complications, the risk posed by ECT to the mother and fetus is minimal. Common fetal adverse events reported in the literature include abortion/stillbirth/neonatal death (4.6%), preterm delivery/preterm labor (4.4%), fetal bradycardia or other arrhythmias (4.2%), and congenital anomalies (3.96%), respectively. In our study, spontaneous abortion occurred in four patients and one infant that was delivered by cesarean section developed neonatal respiratory distress. Literature reviews suggest that neonatal respiratory distress is the least common event (0.36%) in such patients [22]. This event may be considered to be associated with cesarean section. As in previous studies, abortion was the most frequent fetal adverse event in our study. These patients were in the first trimester and three of the four abortions were terminated upon the request of the patients. In our study, the patients were monitored with tocometry for uterine activity. None of our patients had a preterm delivery or preterm labor.

The role of FHR monitoring during non-obstetric procedures is not well-defined. It is generally accepted that in the previable period, auscultation of the fetal heart rate before and after a procedure is acceptable. However, after 24 weeks, when fetal intervention is possible (i.e. cesarean delivery and subsequent neonatal resuscitation), intraoperative fetal monitoring in the setting of non-obstetric procedures during pregnancy can be considered per the American College of Obstetricians and Gynecologists suggestion [17,24]. In our study. FHR was monitored immediately before and immediately after each ECT procedure with a Doppler monitor. We did not detect fetal bradycardia or other arrhythmias in any fetus.

Limitation of the study

Although the number of cases evaluated in our study was higher than those reported in other studies, our study was limited since we evaluated 15 cases over a one-year period, which may not be sufficient for drawing definitive conclusions.

## Conclusions

In conclusion, the combined use of rocuronium, sugammadex, and propofol enables the rapid recovery of the neuromuscular blockage after the ECT procedure. Therefore, anesthetic management during ECT can be performed safely by using propofol and rocuronium with sugammadex in pregnant patients in the postoperative period. In our patients, the combined use of rocuronium, sugammadex, and propofol resulted in no complication in the mothers or fetuses during the short-term follow-up. In the long-term follow-up, however, abortion and neonatal respiratory distress were observed, particularly in the first trimester. A placental anomaly was the most common complication encountered during the third trimester. However, we could not determine whether these complications were caused by the drugs used in the treatment, the long-term effects of ECT, or the anesthetic approach we used. Extensive studies are needed to elucidate this controversy. Moreover, physicians should be aware of the potential maternal and fetal complications associated with ECT, and it is advisable to inform the patient's family in detail before the ECT procedure.
